# Ambivalence Predicts Symptomatology in Cognitive-Behavioral and Narrative Therapies: An Exploratory Study

**DOI:** 10.3389/fpsyg.2019.01244

**Published:** 2019-05-29

**Authors:** Cátia Braga, António P. Ribeiro, Inês Sousa, Miguel M. Gonçalves

**Affiliations:** ^1^ Department of Applied Psychology, Psychology Research Center (CIPsi), University of Minho, Braga, Portugal; ^2^ Department of Mathematics and Applications, Center of Mathematics University of Minho, Braga, Portugal

**Keywords:** ambivalence, ambivalence coding system, ambivalence resolution, poor outcome predictors, innovative moments

## Abstract

**Background:** The identification of poor outcome predictors is essential if we are to prevent therapeutic failure. Ambivalence – defined as a conflictual relationship between two positions of the self: one favoring change and another one favoring problematic stability – has been consistently associated with poor outcomes. However, the precise relationship between ambivalence and clients’ symptomatology remains unclear.

**Objective:** This study aims at assessing ambivalence’s power to predict symptomatology, using a longitudinal design.

**Methods:** The complete 305 sessions of 16 narrative and cognitive-behavioral cases have been analyzed with the Ambivalence Coding System and outcome measures have been used for each session.

**Results:** Ambivalence emerged as a significant predictor of subsequent symptomatology suggesting that ambivalence is not only related to treatment outcomes, but that it represents a strong predictor of subsequent symptomatology.

**Discussion:** The implications of ambivalence’s power to predict outcomes for research and clinical practice are discussed.

While research has revealed the efficacy of psychotherapy in dealing with a variety of psychological problems (e.g., Wampold and Imel, 2015; Cuijpers et al., 2016; Karyotaki et al., 2016), studies have consistently revealed that around 50% of clients experience no change in psychotherapy (Lambert, 2007), about 20% of clients abandon the process prematurely (Leahy, 2012; Swift and Greenberg, 2012), and 5–10% of clients present some level of deterioration (Lambert and Ogles, 2004). In this context, the study of the factors that may contribute to non-improvement and deterioration assumes utmost importance.

When reflecting upon these factors, the concept of resistance assumes unavoidable clinical and empirical significance as “one of the most crucial, pointing toward perhaps the single most important factor—or, more accurately, set of factors—in determining the success or failure of the therapeutic enterprise” (Wachtel, 1999, p. 103). In fact, a robust body of research suggests that higher levels of resistance are consistently associated with poor therapy outcomes and premature treatment termination (see Beutler et al., 2001 for a review), supporting the need for empirical studies that aim at understanding the specific relationship between resistance and therapeutic failure. Resistance can be defined as a set of behaviors that hinders the progress toward desired changes (Beutler et al., 2002, 2011; Leahy, 2012) and may assume different features in distinct therapeutic models. However, most clients do not simply resist change but are hesitant or ambivalent about change so we often observe movements away from and movements toward change in resistant clients. Thus, many of the aspects that are conceptualized as resistance are probably better understood as ambivalence (Engle and Arkowitz, 2008).

Ambivalence is a common human experience and involves simultaneously evaluating an attitude object both in a strong negative and a strong positive way (Kaplan, 1972). Attitude ambivalence is often experienced as unpleasant as it involves the simultaneous accessibility of conflicting thoughts or feelings. Studies have revealed that this may be related to the anticipation of negative emotions – like guilt, fear, disappointment, and regret – that may arise in the wake of a “wrong” decision (see Van Harreveld et al., 2009 for a review).

Distinct consequences have been associated with attitude ambivalence. For example, it involves systematic information processing (Rydell et al., 2008) – which has been argued to serve as a way to reduce anxiety in the face of uncertainty (Maio et al., 1996; Jonas et al., 1997). Attitude ambivalence has also been shown to be more pliable (Bassili, 1996; Armitage and Conner, 2000), to have lower memory accessibility (Bargh et al., 1992), and to be less predictive of behavior (Armitage and Conner, 2000; Sparks et al., 2004).

In psychotherapy, ambivalence involves simultaneous movements toward and away from change – as an approach-avoidance conflict (Dollard and Miller, 1950) – a conflict of the self that, if not properly solved, tends to negatively impact treatment (Miller and Rollnick, 2002; Braga et al., 2016, 2018). Ambivalence – and the importance of its resolution so that real change can be attained – assumed a significant role in clinical practice and research with the Stages of Change Model (Prochaska and DiClemente, 1983; DiClemente and Prochaska, 1985). This model also contributed to the development of Motivational Interviewing (Miller and Rollnick, 1991; DiClemente, 1999), which was designed to deal with ambivalence toward change.

In process research, the acknowledgment of ambivalence’s central role in the process of psychotherapeutic change stirred the development of an empirical marker – the ambivalence marker (AM, previously termed return to the problem marker, Gonçalves et al., 2009) – that allowed for the processual study of ambivalence. AMs are present when after the occurrence of an innovative moment (that is, a novelty or an exception to the maladaptive pattern, see Gonçalves et al., 2017) in the therapeutic dialogue, clients immediately attenuate the meaning of the novelty through a return to the problematic pattern. For example, if a given client’s problematic pattern is characterized by passiveness and submissiveness to others, the following sentence would be coded as an IM: “I do not care what she thinks anymore, I have to tell her how I feel, my feelings matter!” However, if the client continued by saying: “But I’m afraid that I will be feeling very guilty and ridiculous after I say it,” this last sentence would be coded as an ambivalence marker.

Studies that tracked AMs along treatment revealed that in unchanged cases AMs’ frequency is higher – keeping stable or even increasing as the psychotherapeutic process evolves – while for recovered cases the frequency of AMs is generally lower and decreases as treatment progresses (Gonçalves et al., 2011b; Ribeiro et al., 2014, 2015; Alves et al., 2015). Overall, these results suggest that (1) ambivalence – as measured by AMs – is a frequent process both in unchanged and recovered cases; and (2) its persistence along treatment is associated with therapeutic failure. In fact, as change typically involves abandoning entrenched and problematic functioning patterns, ambivalence may, on the one hand, represent a natural “*byproduct of the process of changing complex behaviors*” (Moyers and Rollnick, 2002, p. 187). However, on the other hand, if successful therapy is to take place, the inner conflict expressed by ambivalence must also be properly addressed and overcome (Braga et al., 2016, 2018). This is in line with the argument that ambivalence may constitute not only a hindrance but also an opening for change (Mahalik, 2001), providing that it is effectively dealt with and overcome during therapy (Wachtel, 1999; Braga et al., 2016, 2018; Westra and Norouzian, 2018).

As previously mentioned, studies with the Ambivalence Coding System (Gonçalves et al., 2009, 2017) have been revealing that AMs are associated with poor outcomes. These studies used various samples with different clinical problems and distinct therapeutic models. Yet, most of these models shared a predominantly constructivist or phenomenological approach such as narrative therapy (Ribeiro et al., 2015), meaning reconstruction approach to grief (Alves et al., 2015), and emotion-focused therapy (Ribeiro et al., 2014). Thus, the present study firstly aims at contrasting ambivalence between a sample of narrative therapy and a sample of cognitive-behavioral therapy. Also, studies with AMs (Gonçalves et al., 2011b; Ribeiro et al., 2014, 2015; Alves et al., 2015) have suggested that persistent ambivalence is in some way related to unsuccessful outcomes. However, the precise relationship between AMs and clients’ symptomatology remains unclear as all previous studies associated AMs with pre-post change. In this context, using a longitudinal design, the present study aims at evaluating ambivalence’s power to predict outcomes, assessing the relationship between AMs and outcomes on a session-to-session basis.

As Lambert (2007) advises, preventing therapeutic failure demands the ability to predict poor outcomes. In general, studies conducted by Lambert and collaborators have been revealing that clients’ levels of distress are able to predict deterioration (Lambert et al., 2002; Hannan et al., 2005; Ellsworth et al., 2006; Lutz et al., 2006; Spielmans et al., 2006). In this vein, other variables have been examined such as clients’ dropout, non-adherence, and resistance (e.g., Beutler et al., 2011; De Panfilis et al., 2012; Taylor et al., 2012). The current study adds to this literature by investigating AMs’ impact on subsequent symptoms.

## Materials and Methods

### Samples

The sample of the present study is composed of 16 cases conducted with cognitive-behavioral therapy (CBT) (*n* = 6) and narrative therapy (NT) (*n* = 10) for depression. In the NT sample, seven clients were female and three were male and were, at the time of the study, an average of 41 years old (SD = 14.97). In the CBT sample, five clients were female and one was a male and were an average of 34 years old (SD = 8.48). Both the NT and the CBT samples had integrated a clinical trial (Lopes et al., 2014). All clients had been diagnosed with major depression according to the DSM-IV-TR (American Psychiatric Association, 2000), agreed to have their sessions recorded, and had provided a written informed consent. Clients with: (1) any axis II disorder; (2) any other axis I disorder constituting the central focus of clinical work; (3) severe suicidal ideation; (4) psychotic symptoms; and (5) bipolar disorder were not included in the study. Psychotherapy was delivered individually: nine clients completed 20 sessions, three clients completed 19 sessions, one client completed 18 sessions, one client completed 16 sessions, one completed 15 sessions, and one client completed 12 sessions. Differentiation of recovered and unchanged cases was computed in accordance with a RCI (Jacobson and Truax, 1991) of the BDI-II (McGlinchey et al., 2002). The 16 clients were selected from the wider sample randomly (recovered and unchanged cases balanced) for process research purposes.

Various process research projects have previously analyzed this sample (see Gonçalves et al., 2017 for a description of the studies). In what relates to AMs specifically, a study by Ribeiro et al. (2015) analyzed the NT sample for the association between AMs and treatment outcome (measured by pre-post change). This is the first study to analyze AMs in the CBT sample and to analyze AMs’ power to predict outcomes longitudinally.

### Therapy and Therapist

The CBT group followed the CBT treatment manual for depression (Rush et al., 1977; Beck et al., 1979). The NT manual (Gonçalves and Bento, 2008) was specially developed for Lopes et al. (2014) study and is based on the work of Michael White (White and Epston, 1990; White, 2007). Adherence to the manual and therapist competence were monitored through weekly supervisions (using session’s audiovisual material) and assessed by external judges (see Lopes et al., 2014).

Two therapists integrated the study: one for the CBT and another one for the NT sample. The CBT therapist was a PhD student with 3 years of experience as a cognitive-behavioral psychotherapist. A senior CBT therapist offered weekly supervision and ensured adherence to the CBT model of intervention. The NT therapist had a PhD in clinical psychology and 7 years of clinical practice – three in NT – and was trained in the intervention manual specifically designed for the study, which was inspired on the work of White and Epston (1990).

### Process Measures

#### The Innovative Moments Coding System

The Innovative Moments Coding System (IMCS) allows for the identification of exceptions to the clients’ problematic pattern (Gonçalves et al., 2011a). All sessions had been previously coded with the IMCS by previous studies. Results of this coding can be found in Gonçalves et al. (2016a) for NT and in Gonçalves et al. (2016b) for CBT. The agreement between the two independent judges on overall IM proportion was 0.90 in the CBT sample and 0.89 in the NT sample, with Cohen’s kappa values of 0.94 and 0.91, respectively, revealing strong agreements between judges.

#### Ambivalence Coding System

The Ambivalence Coding System (ACS) allows for the identification of ambivalence markers, that is, the immediate reoccurrence of the problematic pattern after an IM (Gonçalves et al., 2009). The ACS was applied to all sessions of the NT sample in the context of a previous study (Ribeiro et al., 2015), with a Cohen’s Kappa of 0.91, and to all the sessions of the CBT sample in the context of the present study – with a Cohen’s Kappa of 0.94. Both values reveal strong inter-rater agreements.

### Outcome Measures

#### Outcome Questionnaire-10.2

Clients from both samples filled in the Outcome Questionnaire-10.2 (OQ-10.2) at the beginning of every session (Lambert et al., 2005). The OQ 10.2 is a 10-item (rated on a 5-point Likert scale) questionnaire that measures symptomatic change – higher scores indicate higher distress levels. Adequate values of internal consistency and test retest reliability have been demonstrated.

### Analyses

#### Hierarchical Linear Modeling Analyses

A HLM analyzed the longitudinal association between AMs (predictors) and outcomes (OQ-10.2, filled in by clients at the beginning of every session) as a response variable. The model aimed at testing the hypothesis that AMs are able to predict OQ-10-2 scores in the next session (OQ-10-2 score at lag +1) both for NT and CBT. As AMs constitute a proportion of IMs (the IMs that are immediately followed by a return to the problematic pattern), IMs’ proportion was also inserted in the model so we could understand if the impact of AMs on outcomes was still significant when IMs’ proportion was taken into account. Treatment (NT or CBT) was also inserted as a predictor variable in this model. HLM is particularly appropriate for the analysis of nested or hierarchically structured data as is the case in the present study – data collected in different sessions were nested within each client. As HLM allows for effects estimation of both within-clients and between-clients (Woltman et al., 2012), HLM was fitted into a regression model with two hierarchies: (1) within-clients – outcomes estimated to be a function of time – and between-clients.

#### Generalized Linear Mixed-Effects Modeling Analyses

As AMs represent a proportion of IMs, GLMM was used to assess the longitudinal association between symptomatology (OQ10.2 at the beginning of each session) as predictor and AMs as a response variable (i.e., to reverse the prediction direction). This is because GLMM is a type of regression that allows response variables with arbitrary distributions – as is the case with proportions (McCullagh and Nelder, 1989). Thus, A GLMM was fitted, taking into account a subject-specific random effect, assuming variability among individuals, and considering symptomatology (OQ-10.2 score) in each session as predictor of the proportion of AMs in the following session. Generalized linear mixed models (lme4) package for R (Version 3.2.4, R Development Core Team, 2016) was used to perform the analyses.

## Results

As mentioned earlier, AMs represent an immediate return to the problematic pattern after the occurrence of an innovative moment. Thus, AMs are computed as the percentage of IMs – from the total universe of IMs – that constitute AMs. For unchanged cases, the mean percentage of AMs was 15.53% in the first session and 10.91% in the last session. For recovered cases, the mean percentage of AMs in the first session was 17.32%, while in the last session the mean percentage of AMs was 4.11%.

### AMs and IMs as Predictors of Symptoms

AMs emerged as a significant predictor of symptoms in the subsequent session (*p* = 0.009; *R^2^_adj_* = 0.665) (Table 1). Hence, AMs were positively associated with symptomatology (OQ-10.2) in the subsequent session, meaning that lower ambivalence in a given session was associated with lower symptomatology in the next session. IMs were also a significant predictor of symptoms in the next session (*p* < 0.0001; *R^2^_adj_* = 0.665), but IMs negatively associated with symptomatology. Thus, a higher proportion of IMs in one session was associated with lower symptomatology (OQ-10.2) in the subsequent session. Treatment was not a significant predictor (*p* = 0.253; *R^2^_adj_* = 0.665), meaning that the association found between AMs and symptoms was the same for NT and CBT.

**Table 1:** HLM with treatment condition (NT or CBT), IMs’ proportion, and the proportion of AMs as predictors of symptomatology (OQ 10.2 scores) in the next session.

Models and fixed effects	Coefficient	SE	*t*	*p*
AMs and IMs predicting OQ-10.2 model				
Intercept	18.892	2.484	7.605	<0.0001
AMs	5.769	2.191	2.633	0.009
IMs	−0.127	0.027	−4.705	<0.0001
Treatment	3.622	3.035	1.193	0.2526

### Symptoms (OQ-10.2) as Predictors of AMs

In order to understand if symptoms exert an impact in the subsequent session’s AMs, a GLMM analysis was performed – as AMs are computed as a proportion, the use of a regular HLM is impeded (Table 2). Symptomatology (OQ-10.2) emerged as a significant predictor of AMs in the subsequent session (*p* = 0.009; *R^2^_adj_* = 0.060). Treatment was not a significant predictor (*p* = 0.253; *R^2^_adj_* = 0.060), which means that the association between symptomatology (OQ-10.2) and AMs in the subsequent session was the same for NT and CBT.

**Table 2:** GLMM with treatment condition (NT, CBT) and symptomatology (OQ 10.2) predicting AMs proportion in the subsequent session.

Models and fixed effects	Coefficient	SE	*z*	*p*
OQ-10.2 predicting AMs model				
Intercept	−1.982	0.204	−9.715	<0.0001
Treatment	0.302	0.218	1.388	0.1652
OQ10	0.021	0.006	3.594	0.0003

## Discussion

While former studies analyzed the relationship between AMs and pre-post change (Gonçalves et al., 2011b; Ribeiro et al., 2014, 2015; Alves et al., 2015), this study examined the predictive effect of AMs on symptomatic change in the subsequent session. Ribeiro et al. (2015) studied the narrative subsample, integrated here with the CBT subsample, and found a similar proportion of AMs at the beginning of therapy and a decreasing tendency of these markers along the treatment for both unchanged and recovered cases. However, as expected, recovered cases revealed a more pronounced reduction when compared to unchanged cases, suggesting that in recovered cases ambivalence tended to be resolved, while it remained problematic in unchanged cases.

In the present study, we expanded former studies by carrying out a longitudinal design, testing the relationship between ambivalence (AMs) and symptoms’ improvement (OQ-10.2) (Lambert et al., 2005) with two distinct models. One model tested AMs (and IMs) in a given session as predictors of symptoms in the subsequent session, and another model reversed the prediction direction by testing if symptoms in a given session predict AMs in the subsequent session. Results from the former model suggested that IMs and AMs were predictors of symptoms, curiously with similar amount of variance explained. As such, sessions with more IMs were associated with lower symptomology and sessions with lower AMs were also associated with lower symptomatology. The second tested model supports the idea that symptomatology in one session also has an impact on the following session’s ambivalence, in the expected direction (that is, higher symptomatology predicts higher ambivalence in the following session). Thus, results suggest a bidirectional relationship between ambivalence and symptomatology. However, the models also suggested that ambivalence’s ability to predict symptoms in the next session was substantially more adjusted to the data, explaining considerably more variance than the set of models testing the reverse direction. This implies that AMs are not only related to treatment outcomes, but that they represent a strong predictor of posterior symptomatology (i.e., in the next session) – exposing the clinical significance of the ambivalence phenomenon.

As previously mentioned, along with a number of distinct consequences, attitude ambivalence has been linked to systematic processing (Rydell et al., 2008). The authors on social psychology studies have argued that this systematic processing may serve as a way to reduce anxiety in the face of uncertainty (Maio et al., 1996; Jonas et al., 1997). In the context of psychotherapy, ambivalence may constitute a sign that clients are having difficulties progressing in therapy as changing complex and well-settled patterns of functioning often implies a threatening *leap of faith* into the unknown. Also, ambivalence is often an unpleasant state *per se* which seems to relate to the anticipation of negative emotions (see Van Harreveld et al., 2009 for a review) should a “wrong” step be taken – and one could argue this may be one of the routes by which ambivalence relates to treatment outcomes. Ambivalence may trigger other transdiagnostic variables associated with psychopathology – such as rumination (Nolen-Hoeksema and Watkins, 2011) – as a strategy to reduce anxiety, or uncertainty intolerance (Rosser, 2019). In any case, as they signal the probability of the occurrence of a subsequent week characterized by greater psychological suffering, sessions with higher proportion of AMs may be particularly important targets of therapeutic attention.

Dealing with ambivalence requires its understanding in the same intersubjective context in which it occurs – the therapeutic interaction. Although the process of ambivalence has been conceptualized as an intrapersonal process, when it occurs in the therapeutic context, it is not disengaged from the quality of the client-therapist interactive process. Ribeiro et al. (2013) suggest that ambivalent responses from clients may indicate that the therapeutic intervention exceeded the client’s capacity to integrate novelty. In a case study by Ribeiro et al. (2013), the therapist inadvertently stimulated the client’s ambivalence by frequently using challenging interventions after the client expressed ambivalence. *Responsiveness –* defined as “behavior that is affected by emerging context, including emerging perceptions of others’ characteristics and behavior” (Stiles et al., 1998, p. 440) – thus takes a central role when we are dealing with ambivalence in the therapeutic context. Thus, therapists should be able to identify, assess, and appropriately respond to their clients’ ambivalence, balancing supporting, and challenging interventions in a responsive way (see Ribeiro et al., 2013) so as to avoid the promotion of resistance and facilitate the process of change.

Besides attending to moments when clients express ambivalence, therapists should also be alert to potential moments of ambivalence resolution. Studies on ambivalence resolution (Braga et al., 2016, 2018) have identified distinct processes (dominance and negotiation) that are involved in the overcoming of ambivalence. These processes reflect distinct relationships between the two positions that are involved in the ambivalence conflict (favoring change versus favoring problematic stability). In the dominance process, the innovative position strives to regulate the problematic position by affirming the innovative position’s control. In the negotiation process, the conflicting positions are able to communicate with one another, promoting a dynamic flow between opposites, rather than the dominance of one of them (Braga et al., 2016). Retrieving the previously given example of a problematic pattern characterized by passiveness and submissiveness to others, the following sentence exemplifies a dominance type: “I am very clear on this – I will not submit to her will anymore.” In contrast, the following example would be coded as a negotiation type: “It is important for me to feel she is ok with my decision, but I also need to feel this is the right thing for me to do.” These are simple illustrations of what Braga et al. (2016) termed momentary resolutions, that is, “moments when there is an agentic and determined resolution of ambivalence, even if it is a momentary one” (Braga et al., 2016, p. 9). The authors suggest that it is the repetition of these momentary resolutions that allows for the progressive resolution of the conflictual relationship between both positions of the self involved in ambivalence. While both dominance and negotiation exert an impact on ambivalence reduction, negotiation revealed an impact that is nearly five times higher (Braga et al., 2018). Also, negotiation tends to increase from the initial to the final sessions of recovered cases and to be virtually absent in unchanged cases (Braga et al., 2016, 2018), advocating the need for the negotiation and integration between the problematic and the innovative positions of the self involved in ambivalence in order to resolve it. This is consistent with the need for increasing assimilation of problematic experiences proposed by the assimilation model (Stiles, 2002). Thus, therapists should be able to identify and promote ambivalence resolution moments. Particularly, therapists should aim to be responsive to the concerns of both the innovative and the problematic positions of the self – actively avoiding side taking – and promoting moments of communication between the two opposing positions of the self, since the presence of moments of negotiation between the positions has revealed a significant impact on the reduction of ambivalence (Braga et al., 2018).

## Limitations and Conclusion

This study has a diversity of limitations that should be overcome in future studies. Besides the small sample size (although the number of observations is quite significant), part of the sample was previously studied (the NT subsample) on the impact of ambivalence on pre-post change. Also, the low number of therapists prevents the isolation of treatment effects from therapist effects. On the other hand, this study involved the intensive analysis of ambivalence in 305 complete sessions of therapy, which allowed for the study of this process in a highly innovative way. In the same vein, we hope that future studies will balance the necessity of empirical rigor with the need for an in-depth analysis of this phenomenon.

In conclusion, improving treatment results for clients who are predicted to get worse has significant consequences for client care. Although the results from the present study should be taken with caution, if future studies with distinct and larger samples replicate these findings, ambivalence – as measured by the ambivalence marker – may constitute a transtheoretical, significant, and easily detectable aspect of the therapeutic process that therapists may use both as a signal of their clients’ difficulty to integrate novelty and as a developmental opportunity to facilitate the process of change.

## Ethics Statement

Every participant has previously agreed to the recording of the sessions and to the use of this material for research purposes and has been informed of the confidential nature of the information provided. The sample collecting procedures have been approved by the Conselho de Ética of University of Minho.

## Author Contributions

CB implemented the study and developed the theory. AR and MG conceived the concept of AMs, encouraged the investigation of ambivalence’s role as a predictor of symptoms, and supervised the findings of this work. IS performed the computations and supervised the result’s description. All authors discussed the results and contributed to the final manuscript.

### Conflict of Interest Statement

The authors declare that the research was conducted in the absence of any commercial or financial relationships that could be construed as a potential conflict of interest.

## References

[ref1] Alves D. Fernández-Navarro P. Ribeiro A. P. Ribeiro E. Sousa I. Gonçalves M. M. (2015). Ambivalence in grief therapy: the interplay between change and self-stability. Death Stud. 40, 129–138. 10.1080/07481187.2015.1102177 26466743

[ref3] American Psychiatric Association (2000). DSM-IV-TR. (Lisboa: CLIMEPSI).

[ref4] Armitage C. J. Conner M. (2000). Attitudinal ambivalence: a test of three key hypotheses. Personal. Soc. Psychol. Bull. 26, 1421–1432. 10.1177/0146167200263009

[ref5] Bargh J. A. Chaiken S. Govender R. Pratto F. (1992). The generality of the automatic attitude activation effect. J. Pers. Soc. Psychol. 62, 893–912. 10.1037/0022-3514.62.6.893, PMID: 1619549

[ref6] Bassili J. N. (1996). Meta-judgmental versus operative indexes of psychological attributes: the case of measures of attitude strength. J. Pers. Soc. Psychol. 71, 637–653. 10.1037/0022-3514.71.4.637

[ref7] Beck A. T. Rush A. J. Shaw B. F. Emery G. (1979). Cognitive therapy of depression. (New York: Guildford Press).

[ref9] Beutler L. E. Harwood T. M. Michelson A. Song X. Holman J. (2011). Resistance/reactance level. J. Clin. Psychol. 67, 133–142. 10.1002/jclp.20753, PMID: 21108314

[ref10] Beutler L. E. Moleiro C. Talebi H. (2002). Resistance in psychotherapy: what conclusions are supported by research. J. Clin. Psychol. 58, 207–217. 10.1002/jclp.1144, PMID: 11793333

[ref11] Beutler L. E. Rocco F. Moleiro C. M. Talebi H. (2001). Resistance. Psychother. Theory Res. Pract. Train. 38, 431–436. 10.1037/0033-3204.38.4.431

[ref12] Braga C. Oliveira J. T. Ribeiro A. P. Gonçalves M. M. (2016). Ambivalence resolution in emotion-focused therapy: the successful case of Sarah. Psychother. Res. 28, 423–432. 10.1080/10503307.2016.1169331 27196812

[ref13] Braga C. Ribeiro A. P. Gonçalves M. M. Oliveira J. T. Botelho A. Ferreira H.. (2018). Ambivalence resolution in brief psychotherapy for depression. Clin. Psychol. Psychother. 25, 369–377. 10.1002/cpp.2169, PMID: 29316007

[ref14] Cuijpers P. Donker T. Weissman M. M. Ravitz P. Cristea I. A. (2016). Interpersonal psychotherapy for mental health problems: a comprehensive meta-analysis. Am. J. Psychiatr. 173, 680–687. 10.1176/appi.ajp.2015.15091141, PMID: 27032627

[ref15] De Panfilis C. Marchesia C. Cabrinob C. Monicib A. Politib V. Rossib M.. (2012). Patient factors predicting early dropout from psychiatric outpatient care for borderline personality disorder. Psychiatry Res. 200, 422–429. 10.1016/j.psychres.2012.03.016, PMID: 22503328

[ref16] DiClemente C. C. (1999). Motivation for change: implications for substance abuse. Psychol. Sci. 10, 209–213. 10.1111/1467-9280.00137

[ref17] DiClemente C. C. Prochaska J. O. (1985). “Processes and stages of change: coping and competence in smoking behavior change” in Coping and substance use: A conceptual framework. eds. Shiffman S. Wills T. (New York: Academic Press).

[ref18] Dollard J. Miller N. E. (1950). Personality and psychotherapy: An analysis in terms of learning, thinking, and culture. (New York: McGraw-Hill).

[ref19] Ellsworth J. R. Lambert M. J. Johnson J. (2006). A comparison of the outcome questionnaire-45 and outcome questionnaire-30 in classification and prediction of treatment outcome. Clin. Psychol. Psychother. 13, 380–391. 10.1002/cpp.503

[ref20] Engle D. Arkowitz H. (2008). Viewing resistance as ambivalence: integrative strategies for working with resistant ambivalence. J. Humanist. Psychol. 48, 389–412. 10.1177/0022167807310917

[ref21] Gonçalves M. M. Bento T. (2008). Manual terapêutico psicoterapia narrativa de re-autoria. (Portugal: Braga).

[ref26] Gonçalves M. M. Matos M. Santos A. (2009). Narrative therapy and the nature of “innovative moments” in the construction of change. J. Constr. Psychol. 22, 1–23. 10.1080/10720530802500748

[ref24] Gonçalves M. M. Ribeiro A. P. Mendes I. Alves D. Silva J. Rosa C.. (2017). Three narrative-based coding systems: innovative moments, ambivalence and ambivalence resolution. Psychother. Res. 27, 270–282. 10.1080/10503307.2016.1247216, PMID: 27855544

[ref25] Gonçalves M. M. Ribeiro A. Mendes I. Matos M. Santos A. (2011a). Tracking novelties in psychotherapy process research: the innovative moments coding system. Psychother. Res. 21, 497–509. 10.1080/10503307.2011.560207 21480054

[ref27] Gonçalves M. M. Ribeiro A. P. Silva J. Mendes I. Sousa I. (2016a). Narrative innovations predict symptom improvement: studying innovative moments in narrative therapy of depression. Psychother. Res. 26, 425–435. 10.1080/10503307.2015.1035355 25968420

[ref28] Gonçalves M. M. Ribeiro A. P. Stiles W. B. Conde T. Santos A. Matos M. (2011b). The role of mutual in-feeding in maintaining problematic self-narratives: exploring one path to therapeutic failure. Psychother. Res. 21, 27–40. 10.1080/10503307.2010.507789 20981626

[ref29] Gonçalves M. M. Silva J. R. Mendes I. Rosa C. Ribeiro A. P. Batista J. (2016b). Narrative changes predict a decrease in symptoms in CBT for depression: an exploratory study. Clin. Psychol. Psychother. 24, 836–845. 10.1002/cpp.2048 27766698

[ref30] Hannan C. Lambert M. J. Harmon C. Nielsen S. L. Smart D. W. Shimokawa K.. (2005). A lab test and algorithms for identifying clients at risk for treatment failure. J. Clin. Psychol. 61, 155–163. 10.1002/jclp.20108, PMID: 15609357

[ref31] Jacobson N. S. Truax P. (1991). Clinical significance: a statistical approach to defining meaningful change in psychotherapy research. J. Consult. Clin. Psychol. 59, 12–19. 10.1037/0022-006X.59.1.12, PMID: 2002127

[ref32] Jonas K. Diehl M. Brömer P. (1997). Effects of attitudinal ambivalence on information processing and attitude-intention consistency. J. Exp. Soc. Psychol. 33, 190–210. 10.1006/jesp.1996.1317

[ref33] Kaplan K. J. (1972). On the ambivalence–indifference problem in attitude theory and measurement: a suggested modification of the semantic differential technique. Psychol. Bull. 77, 361–372. 10.1037/h0032590

[ref34] Karyotaki E. Smit Y. Holdt Henningsen K. Huibers M. J. H. Robays J. de Beurs D.. (2016). Combining pharmacotherapy and psychotherapy or monotherapy for major depression? A meta-analysis on the long-term effects. J. Affect. Disord. 194, 144–152. 10.1016/j.jad.2016.01.036, PMID: 26826534

[ref35] Lambert M. J. (2007). Presidential address: what we have learned from a decade of research aimed at improving psychotherapy outcome in routine care. Psychother. Res. 17, 1–14. 10.1080/10503300601032506

[ref38] Lambert M. J. Finch A. Okiishi J. Burlingame G. (2005). Administration and scoring manual for the OQ-10.2. (Salt Lake City, UT: OQ Measures, LLC).

[ref39] Lambert M. J. Ogles B. M. (2004). “The efficacy and effectiveness of psychotherapy” in Bergin and Garfield’s Handbook of psychotherapy and behavior change. 5th edn. ed. Lambert M. J. (New York: Wiley), 139–193.

[ref40] Lambert M. J. Whipple J. L. Bishop M. J. Vermeersch D. A. Gray G. V. Finch A. E. (2002). Comparison of empirically derived and rationally derived methods for identifying clients at risk for treatment failure. Clin. Psychol. Psychother. 9, 149–164. 10.1002/cpp.333

[ref41] Leahy R. L. (2012). Overcoming resistance in cognitive therapy: (New York: Guilford Press).

[ref42] Lopes R. Gonçalves M. M. Machado P. P. Sinai D. Salgado J. (2014). Narrative therapy vs. cognitive-behavioral therapy for moderate depression: empirical evidence from a controlled clinical trial. Psychother. Res. 24, 1–13. 10.1080/10503307.2013.874052 24479576

[ref43] Lutz W. Lambert M. J. Harmon S. C. Stulz N. Tschitsaz A. Schürch E. (2006). The probability of treatment success, failure and duration—what can be learned from empirical data to support decision making in clinical practice? Clin. Psychol. Psychother. 13, 223–232. 10.1002/cpp.496

[ref44] Mahalik J. R. (2001). “Understanding client resistance in therapy: implications from research on the counseling process” in Counseling based on process research: Applying what we know. ed. Tryon G. S. (Boston: Allyn and Bacon), 66–80.

[ref45] Maio G. R. Bell D. W. Esses V. M. (1996). Ambivalence and persuasion: the processing of messages about immigrant groups. J. Exp. Soc. Psychol. 32, 513–536. 10.1006/jesp.1996.0023, PMID: 8979932

[ref46] McCullagh P. Nelder J. (1989). Generalized linear model. (London, UK: Chapman & Hall).

[ref47] McGlinchey J. B. Atkins D. C. Jacobson N. S. (2002). Clinical significance methods: which one to use and how useful are they? Behav. Ther. 33, 529–550. 10.1016/s0005-7894(02)80015-6

[ref49] Miller W. R. Rollnick S. (1991). Motivational interviewing: Preparing people to change addictive behavior. (New York: Guilford Press).

[ref50] Miller W. Rollnick S. (2002). Motivational interviewing: Preparing people for change. 2nd edn. (New York: Guilford Press).

[ref51] Moyers T. B. Rollnick S. (2002). A motivational interviewing perspective on resistance in psychotherapy. J. Clin. Psychol. 58, 185–193. 10.1002/jclp.1142, PMID: 11793331

[ref72] Nolen-Hoeksema S. Watkins E. (2011). A Heuristic for Developing Transdiagnostic Models of Psychopathology Explaining Multifinality and Divergent Trajectories. Perspect. Psychol. Sci. 6, 589–609. 10.1177/1745691611419672, PMID: 26168379

[ref52] Prochaska J. DiClemente C. (1983). Stages and processes of self-change of smoking: toward an integrative model of change. J. Consult. Clin. Psychol. 51, 390–395. 10.1037/0022-006x.51.3.390, PMID: 6863699

[ref53] R Development Core Team (2016). The R Foundation for statistical computing. Retrieved from: http://www.rproject.org

[ref55] Ribeiro A. P. Gonçalves M. M. Silva J. R. Brás A. Sousa I. (2015). Ambivalence in narrative therapy: a comparison between recovered and unchanged cases. Clin. Psychol. Psychother. 23, 166–175. 10.1002/cpp.1945 25808359

[ref56] Ribeiro A. P. Mendes I. Stiles W. B. Angus L. Sousa I. Gonçalves M. M. (2014). Ambivalence in emotion-focused therapy for depression: the maintenance of problematically dominant self-narratives. Psychother. Res. 24, 702–710. 10.1080/10503307.2013.879620, PMID: 24552124

[ref57] Ribeiro E. Ribeiro A. P. Gonçalves M. M. Horvath A. O. Stiles W. B. (2013). How collaboration in therapy becomes therapeutic: the therapeutic collabration coding system. Psychol. Psychother. 86, 294–314. 10.1111/j.2044-8341.2012.02066.x 23955792

[ref74] Rosser B. (2019). Intolerance of Uncertainty as a Transdiagnostic Mechanism of Psychological Difficulties: A Systematic Review of Evidence Pertaining to Causality and Temporal Precedence. Cogn. Ther. Res. 43, 438–463. 10.1007/s10608-018-9964-z

[ref58] Rush A. J. Beck A. T. Kovacs M. Hollon S. D. (1977). Comparative efficacy of cognitive therapy and pharmacotherapy in the treatment of depressed outpatients. Cogn. Ther. Res. 1, 17–37. 10.1007/BF01173502

[ref59] Rydell R. J. McConnell A. R. Mackie D. M. (2008). Consequences of discrepant explicit and implicit attitudes: cognitive dissonance and increased information processing. J. Exp. Soc. Psychol. 44, 1526–1532. 10.1016/j.jesp.2008.07.006

[ref60] Sparks P. Harris P. R. Lockwood N. (2004). Predictors and predictive effects of ambivalence. Br. J. Soc. Psychol. 43, 371–383. 10.1348/0144666042037980, PMID: 15479536

[ref61] Spielmans G. I. Masters K. S. Lambert M. J. (2006). A comparison of rational versus empirical methods in prediction of negative psychotherapy outcome. Clin. Psychol. Psychother. 13, 202–214. 10.1002/cpp.491

[ref62] Stiles W. B. (2002). “Assimilation of problematic experiences” in Psychotherapy relationships that work: Therapist contributions and responsiveness to patients. ed. Norcross J. C. (New York: Oxford University Press), 357–365.

[ref73] Stiles W. B. Honos-Webb L. Surko M. (1998). Responsiveness in Psychotherapy. Psychology: Science and Practice 5, 439–458. 10.1111/j.1468-2850.1998.tb00166.x, PMID: 22440391

[ref63] Swift J. K. Greenberg R. P. (2012). Premature discontinuation in adult psychotherapy: a metaanalysis. J. Consult. Clin. Psychol. 80, 547–559. 10.1037/a0028226 22506792

[ref64] Taylor S. Abramowitz J. S. Mckay D. (2012). Non-adherence and non-response in the treatment of anxiety disorders. J. Anxiety Disord. 26, 583–589. 10.1016/j.janxdis.2012.02.010, PMID: 22440391

[ref65] Van Harreveld F. van der Pligt J. de Liver Y. N. (2009). The agony of ambivalence and ways to resolve it introducing the MAID model. Personal. Soc. Psychol. Rev. 13, 45–61. 10.1177/1088868308324518, PMID: 19144904

[ref66] Wachtel P. L. (1999). Resistance as a problem for practice and theory. J. Psychother. Integr. 9, 103–118. 10.1023/A:1023262928748

[ref67] Wampold B. E. Imel Z. E. (2015). The great psychotherapy debate: The evidence for what makes psychotherapy work: (New York: Routledge).

[ref68] Westra H. A. Norouzian N. (2018). Using motivational interviewing to manage process markers of ambivalence and resistance in cognitive behavioral therapy. Cogn. Ther. Res. 42, 193–203. 10.1007/s10608-017-9857-6

[ref69] White M. (2007). Maps of narrative practice. (New York, NY: WW Norton & Company).

[ref70] White M. Epston D. (1990). Narrative means to therapeutic ends. (New York, NY: WW Norton & Company).

[ref71] Woltman H. Feldstain A. MacKay J. C. Rocchi M. (2012). An introduction to hierarchical linear modeling. Tutor. Quant. Methods Psychol. 8, 52–69. 10.20982/tqmp.08.1.p052

